# NAD-Glycohydrolase Depletes Intracellular NAD^+^ and Inhibits Acidification of Autophagosomes to Enhance Multiplication of Group A *Streptococcus* in Endothelial Cells

**DOI:** 10.3389/fmicb.2018.01733

**Published:** 2018-08-03

**Authors:** Cheng-Lu Hsieh, Hsuan-Min Huang, Shu-Ying Hsieh, Po-Xing Zheng, Yee-Shin Lin, Chuan Chiang-Ni, Pei-Jane Tsai, Shu-Ying Wang, Ching-Chuan Liu, Jiunn-Jong Wu

**Affiliations:** ^1^Institute of Basic Medical Sciences, College of Medicine, National Cheng Kung University, Tainan, Taiwan; ^2^Department of Medical Laboratory Science and Biotechnology, College of Medicine, National Cheng Kung University, Tainan, Taiwan; ^3^Institute of Molecular Medicine, College of Medicine, National Cheng Kung University, Tainan, Taiwan; ^4^Center of Infectious Disease and Signaling Research, National Cheng Kung University, Tainan, Taiwan; ^5^Department of Microbiology and Immunology, College of Medicine, National Cheng Kung University, Tainan, Taiwan; ^6^Department of Microbiology & Immunology, College of Medicine, Chang Gung University, Taoyuan, Taiwan; ^7^Molecular Infectious Disease Research Center, Chang Gung Memorial Hospital, Taoyuan, Taiwan; ^8^Department of Pediatrics, National Cheng Kung University Hospital, College of Medicine, National Cheng Kung University, Tainan, Taiwan; ^9^Department of Biotechnology and Laboratory Science in Medicine, National Yang-Ming University, Taipei, Taiwan

**Keywords:** group A *Streptococcus*, NADase, NAD^+^ balance, acidification, intracellular multiplication

## Abstract

Group A *Streptococcus* (GAS) is a human pathogen causing a wide spectrum of diseases, from mild pharyngitis to life-threatening necrotizing fasciitis. GAS has been shown to evade host immune killing by invading host cells. However, how GAS resists intracellular killing by endothelial cells is still unclear. In this study, we found that strains NZ131 and A20 have higher activities of NADase and intracellular multiplication than strain SF370 in human endothelial cells (HMEC-1). Moreover, *nga* mutants of NZ131 (SW957 and SW976) were generated to demonstrate that NADase activity is required for the intracellular growth of GAS in endothelial cells. We also found that intracellular levels of NAD^+^ and the NAD^+^/NADH ratio of NZ131-infected HMEC-1 cells were both lower than in cells infected by the *nga* mutant. Although both NZ131 and its *nga* mutant were trapped by LC3-positive vacuoles, only *nga* mutant vacuoles were highly co-localized with acidified lysosomes. On the other hand, intracellular multiplication of the *nga* mutant was increased by bafilomycin A1 treatment. These results indicate that NADase causes intracellular NAD^+^ imbalance and impairs acidification of autophagosomes to escape autophagocytic killing and enhance multiplication of GAS in endothelial cells.

## Introduction

Group A *Streptococcus* (GAS) is an important human pathogen responsible for causing wide spectrum of diseases, ranging from superficial infections to life-threatening manifestations including necrotizing fasciitis and streptococcal toxic-shock syndrome (Cunningham, 2008). Although GAS is not considered as intracellular pathogen, increased evidences have shown that GAS can invade epithelial cells to escape killing by host immune responses and antibiotics (LaPenta et al., 1994; Osterlund et al., 1997; Kaplan et al., 2006).

Group A *Streptococcus* expresses numerous virulence factors for subverting host defense mechanisms to successfully establish infection in the host (Barnett et al., 2015). Streptolysin O (SLO) and its cotoxin NAD-glycohydrolase (NADase or SPN) have been reported to be involved in bacterial intracellular survival. SLO is a pore-forming toxin that forms oligomeric pores to disrupt cell membranes and facilitate autophagy formation, which contributes to enhance GAS survival in the intracellular niche of host cells (Sierig et al., 2003; Nakagawa et al., 2004). NADase is encoded by the *nga* gene in the *nga*-*ifs*-*slo* operon (Kimoto et al., 2005), which not only physically interacts but also functionally synergizes with SLO to enhance the cytotoxicity of infected cells (Madden et al., 2001; Bricker et al., 2005; Michos et al., 2006; Velarde et al., 2017). Recent studies also showed that the epidemic M1 and M89 GAS strains, which are rapidly spreading globally, produce higher levels of NADase and SLO to cause severe tissue destruction (Turner et al., 2015; Zhu et al., 2015a,b, 2016), indicating the importance of NADase and SLO in GAS pathogenesis.

Autophagy is a conserved catabolic process that transports cytosolic cargo to lysosomes for maintaining cellular homeostasis in adverse conditions. In addition to metabolic adaptation to nutrient deprivation, autophagy is required for the elimination of intracellular pathogens (Huang and Brumell, 2009; Shahnazari and Brumell, 2011; Deretic et al., 2013; Pareja and Colombo, 2013). In epithelial cells, several studies showed that invading GAS can be targeted into autophagosome-like structures in a SLO-dependent manner. Nonetheless, SLO and NADase prevent trafficking of the GAS-containing vacuole to lysosomes and resulting in delayed intracellular killing (Nakagawa et al., 2004; Amano et al., 2006; Logsdon et al., 2011; O’Seaghdha and Wessels, 2013; Sharma et al., 2016). Our previous study found that insufficient acidification of the autophagosome allows GAS to survive and multiply in endothelial cells, and SLO plays an important role in GAS multiplication (Lu et al., 2015). However, how GAS regulates autophagosomal acidification in endothelial cells is still not clear. In this study, we found that NADase depletes intracellular NAD^+^ storage and inhibits autophagosomal acidification, which is important for promoting intracellular multiplication of GAS in human endothelial cells (HMEC-1).

## Materials and Methods

### Bacteria and Cell Culture Conditions

GAS strains SF370 (M1 serotype) and NZ131 (M49 serotype) were purchased from the American Type Culture Collection (Manassas, VA, United States). GAS strain A20 (M1 serotype) was isolated from a patient with necrotizing fasciitis (Zheng et al., 2013). GAS strains were cultured on tryptic soy agar containing 5% defibrinated sheep blood or tryptic soy broth (Becton Dickinson, Sparks, MD, United States) supplemented with 0.5% yeast extract (TSBY). For genetic manipulation, *Escherichia coli* strain DH5α was cultured in Luria-Bertani (LB) broth (Becton Dickinson). When appropriate, medium was supplemented with antibiotics at the following concentrations: 25 μg/ml of chloramphenicol (Merck, Darmstadt, Germany) for *E. coli*, 3 μg/ml for GAS; 100 μg/ml of spectinomycin (Sigma-Aldrich, St. Louis, MO, United States) for *E. coli* and GAS. Human microvascular endothelial cell line-1 (HMEC-1) cells were cultured in endothelial cell growth medium M200 supplemented with low serum growth factors (Gibco Life Technologies, Grand Island, NY, United States) and 8% fetal bovine serum (FBS) at 37°C in a 5% CO_2_ humidified incubator. Cells were maintained at 0.75 × 10^6^ in 10-cm dish or seeded at 3 × 10^5^ in 6-well plates for intracellular growth analysis and co-localization observation.

### Construction of Isogenic Mutants

To construct the *nga* mutant, 500- and 600-bp fragments of upstream and downstream region of *nga* were amplified by PCR. The PCR fragments containing restriction endonuclease sites (*Eco*RI and *Bam*HI) were ligated with a chloramphenicol cassette and cloned into streptococcal suicide vector pSF152 to generate plasmid pMW790. The recombinant plasmid was subsequently electroporated into strain NZ131 to generate the *nga* mutant (SW957) by homologous recombination. The *nga* mutant was confirmed by Southern blot hybridization and NADase activity. Moreover, the native promoter of *nga* and its structure region (2,175 bp) was amplified by primers *nga*+*ifs-*F/R and cloned into the streptococcal shuttle vector pDL278 for *trans*-complementation (SW958).

The G330D substitution of NADase was constructed by overlap PCR with primers *nga*+*ifs-*F/R and NADase G330D-F/R. The PCR products were digested with restriction endonucleases *Eco*RI and *Bam*HI (New England Biolabs, Hitchin, United Kingdom), and ligated into the temperature-sensitive vector pCN143 (Chiang-Ni et al., 2016) to generate plasmid pMW860. The DNA insert was confirmed by restriction enzyme digestion and DNA sequencing. The native chromosomal locus of NZ131 was exchanged with the mutant allele by allelic exchange when grown at 37°C for insertion and 30°C for excision. Eventually, the NADase G330D mutant (SW976) was verified by DNA sequencing and NADase activity. All oligonucleotide primers are listed in Supplementary Table S1.

### cDNA Preparation and Quantitative RT-PCR

The cDNA synthesis and quantitative RT-PCR protocols were described previously (Wang et al., 2013). Oligonucleotide sequences used for qPCR are listed in Supplementary Table S1. The gyrase subunit A (*gyrA*) of GAS was set as a reference control. The thermocycling reactions were performed in a Lightcycler 2.0 instrument (Roche Diagnostics, Indianapolis, IN, United States) and the crossing point (CP) was analyzed by LightCycler 3.0 software (version 3.0; Roche Diagnostics). The relative gene transcriptional level was calculated following the formula: ratio = 2^∧[ΔCPtarget(control-sample)-^ ^ΔCPreference(control-sample)]^ (Pfaffl, 2001).

### Measurement of NADase Activity

The NADase activity in bacterial culture supernatants was determined by measuring the fluorescence intensity upon excitation with UV light, as described previously (Bricker et al., 2002). Briefly, GAS supernatants from the early stationary phase of liquid cultures were obtained by centrifugation at 2,330 *g* for 10 min. The supernatants were mixed with 1 mM of NAD^+^ (Sigma-Aldrich) in microtiter plates and then incubated at 37°C in 5% CO_2_ for 1 h. To stop the reaction, sodium hydroxide (PanReac AppliChem, Barcelona, Spain) was added to 2 N and reactants were incubated at room temperature in the dark for 1 h. The fluorescence of NAD^+^ was visually read on a Tecan M200 Pro Infinite plate reader (Tecan, Crailsheim, Germany) with a 360-nm excitation. Uninoculated culture medium was added to microtiter plates as a negative control. The NADase activity of each sample was expressed as a relative percentage compared with the negative control.

### Intracellular GAS Multiplication Analysis

Group A *Streptococcus* was cultured in TSBY for overnight and transferred to fresh broth at 1:50 dilution, and then incubated at 37°C in 5% CO_2_ to mid-logarithmic phase. Bacteria were collected by centrifugation at 2,330 *g* for 10 min, washed with PBS, and resuspended in M200 medium containing 8% FBS. HMEC-1 cells were seeded at 3 × 10^5^ in 6-well plates for 18 h, washed with PBS and infected with GAS. In order to achieve same intracellular bacterial load (1.5 × 10^5^ cfu/well) at 1 h of infection, HMEC-1 cells were infected with serotype M1 SF370 and A20 at multiplicity of infection (M.O.I.) of 10, whereas HMEC-1 cells infected with serotype M49 NZ131 at M.O.I of 1. The plates were centrifuged at 500 *g* for 5 min at room temperature to ensure GAS adherence to the cell surface, and then incubated at 37°C in 5% CO_2_ for 30 min. After infection, cells were washed with PBS and treated with 125 μg/ml of gentamicin to kill extracellular bacteria at 37°C in 5% CO_2_ for 1 h (T1). After 1 h treatment, cells were washed and maintained in antibiotic-free medium for an additional 2 h (T3) and 4 h (T5). To calculate the multiplication of intracellular GAS, cells were lysed by deionized water for 5 min, and then plated on TSBY plates to count the number of CFU at various periods of time as indicated in each experiment.

### NAD^+^ Quantification

Intracellular NAD^+^ level of HMEC-1 cells was determined by the NAD^+^/NADH quantification kit (Sigma-Aldrich) following the manufacturer’s instructions. Briefly, infected-HMEC-1 cells were washed with PBS and detached by trypsin-EDTA (Gibco Life Technologies) after infection. Cells (2 × 10^5^) were resuspended in NADH/NAD^+^ extraction buffer and disrupted by freezing in liquid nitrogen and thawing at room temperature for three cycles. The extracts were clarified by centrifugation at 13,000 *g* for 10 min and cellular NADH consuming enzymes were then removed by filtering through a 10-kDa cut-off spin column (GE Healthcare, Buckinghamshire, United Kingdom). For detection of intracellular NADH level, 200 μl of extracted samples were heated at 60°C for 30 min to decompose NAD^+^ and then transferred to a microtitre plate. To determine the total NADH level, 50 μl of duplicate extraction samples were arrayed in microtitre plates. The NAD cycling buffer containing NAD cycling enzyme was added to each well to convert NAD^+^ to NADH. After incubation, each sample was mixed with NADH developer and incubated at room temperature for 1 h. The absorbance of plate wells was measured at 450 nm with a Tecan M200 Pro Infinite microplate reader (Tecan). The NAD^+^/NADH ratio was calculated following a formula: [(NAD_total_ – NADH) / NADH].

### MTS Cell Viability Assay

To determine cell viability of infected endothelial cells, the metabolic activity of mitochondria was measured by the MTS assay according to the manufacturer’s instructions (Promega, Madison, WI, United States). Briefly, cells were infected with GAS as described above, and then fresh M200 medium containing MTS tetrazolium reagent was added to each well at indicated time point. After 2 h of incubation, the absorbance was measured at 490 nm by the Tecan M200 Pro Infinite microplate reader (Tecan). The cell viability of each well was expressed as a relative percentage compared with non-infected cells.

### Confocal Microscopy

HMEC-1 cells were seeded at 3 × 10^5^ in 6-well plates containing a collagen-coated cover slide (23 × 23-mm) and incubated at 37°C in 5% CO_2_ for 18 h. Cells were infected with GAS as described above. For acidification labeling, cells were stained with medium M200 containing 200 nM of lysotracker (red DND-99; Invitrogen Molecular Probes, Eugene, OR, United States) for 2 h after infection. At the indicated times, cells were fixed with 4% paraformaldehyde, permeabilized with 0.4% Triton X-100, and blocked with 1% bovine serum albumin (BSA). After treatments, anti-LC3 (Medical and Biological Laboratories, Nagoya, Japan) and anti-LAMP (Cell Signaling Technology, Danvers, MA, United States) primary antibodies with an appropriate working concentration were added to the cells at 4°C for overnight. Finally, cells were incubated with Alexa Fluor-conjugated secondary antibodies and DAPI at room temperature in the dark, and then mounted with mounting medium (Vector Laboratories, Burlingame, CA, United States) onto a microscope slide and stored at 4°C in the dark prior to imaging. Intracellular co-localization was analyzed by confocal laser scanning microscopy (FV1000; Olympus, Tokyo, Japan). Bacteria co-localization with the lysosomal marker LAMP1 and a low-pH indicator were quantified from five independent fields of three separate experiments.

### Western Blot Analysis

Total proteins were harvested from cell lysate with RIPA lysis buffer containing protease inhibitor (Promega) and separated by SDS-polyacrylamide gel. Proteins were transferred to the PVDF membrane and hybridized with primary antibodies anti-LC3. Subsequently, secondary horseradish peroxidase (HRP)-conjugated goat anti-rabbit antibodies (Jackson Immunoresearch Laboratories, West Grove, PA, United States) were used at 1:10,000 dilution and the signal was visualized with an ImageQuant LAS-4000 mini (GE Healthcare).

### Statistical Analysis

Data represent the mean values and standard error of the mean from at least three independent experiments. GraphPad Prism 5.0 (GraphPad Software) was used for all statistical analyses. The statistical significance of differences between independent experiments was evaluated using the one-way or two-way ANOVA with *post hoc* Tukey’s or Bonferroni multiple comparison test, respectively. *p*-values of less than 0.05 were considered statistically significant and were indicated by an asterisk (^∗^) in figures.

## Results

### The Enzymatic Activity of NADase Is Required for the Intracellular Multiplication of GAS in HMEC-1 Cells

**FIGURE 1:**
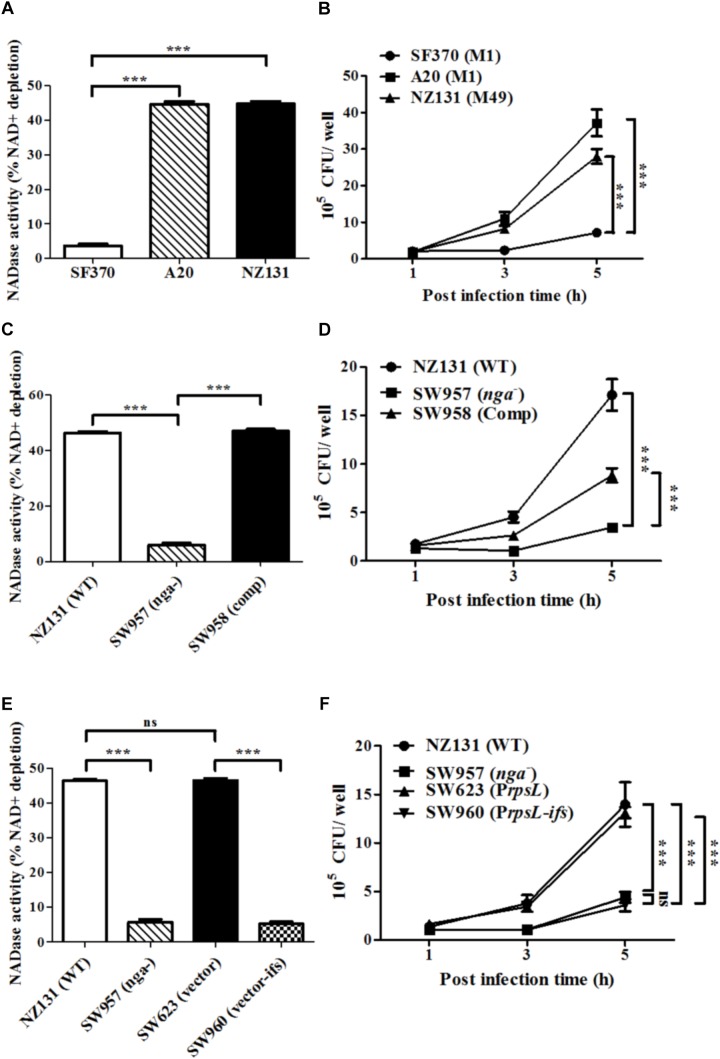
NADase contributes to intracellular multiplication of GAS in endothelial cells. **(A)** NADase activity of A20, SF370, and NZ131. NADase activity was determined by co-incubation with NAD^+^ and expressed as relative percentage compared to medium alone. **(B)** HMEC-1 cells were infected with serotype M1 SF370 and A20 (M.O.I. = 10), or M49 NZ131 (M.O.I. = 1) for 30 min, and treated with gentamicin to kill extracellular bacteria. Intracellular viable bacteria were counted by CFU-based assays. **(C)** NADase activity was determined by co-incubation with NAD^+^ and expressed as relative percentage compared to medium alone. **(D)** Wild-type NZ131 and its isogenic strains infected HMEC-1 cells at M.O.I. of 1 and intracellular viable bacteria were counted by CFU-based assays. **(E)** NADase activities of NZ131, *nga* mutant, *ifs*-overexpressing, and vector control strains were determined by co-incubation with NAD^+^ and expressed as relative percentage compared to medium alone. **(F)** HMEC-1 cells were infected with *ifs*-overexpressed NZ131 at M.O.I. of 1 and intracellular viable bacteria were counted by CFU-based assays. The data represent the means ± SEM of at least three independent experiments. ^∗∗∗^*p* < 0.001 (one- or two-way ANOVA).

NADase has been shown to enhance GAS intracellular survival in epithelial cells (O’Seaghdha and Wessels, 2013; Chandrasekaran and Caparon, 2015; Sharma et al., 2016). However, the role of NADase in GAS survival in endothelial cells has yet to be explored. To investigate whether NADase is involved in GAS survival in endothelial cells, the NADase activity in bacterial supernatants from GAS strains SF370, A20 and NZ131 was measured. Moreover, HMEC-1 cells infected with strains SF370, A20 and NZ131 were analyzed by the gentamicin protection assay. The results showed that strains A20 and NZ131 have better NADase activity, and an increased number of intracellular bacteria after 5 h of infection, compared to strain SF370 (**Figures 1A,B**).

To elucidate whether NADase contributes to GAS multiplication in endothelial cells, the *nga* mutant of NZ131 (SW957) was constructed. The results showed that deletion of *nga* abolishes its NADase activity (**Figure 1C**). Since *nga*-*ifs*-*slo* is an operon in GAS (Kimoto et al., 2005), the transcription of each gene in the *nga* mutant was evaluated by qRT-PCR. Results showed that the expression of *ifs* and *slo* are not affected by the *nga* deletion (Supplementary Figures S1A–C). NADase has been shown to have a toxic effect for bacteria (Meehl et al., 2005; Kimoto et al., 2006). In order to avoid NADase toxicity, intact *nga*-*ifs* expression was generated in the *nga*-complementary strain (SW958) (Supplementary Figures S1A–C). Next, HMEC-1 cells were infected with wild-type NZ131 and its isogenic strains. Inactivation of *nga* resulted in about 5-fold and 3-fold decreases in the intracellular bacterial load in HMEC-1 cells after 5 h of infection, compared to wild-type NZ131 and the *nga* complemented strain SW958, respectively (**Figure 1D**). To exclude that the difference in intracellular multiplication of GAS was due to differences in internalization efficiency, the number of cell associated and internalized bacteria was enumerated after 30 min of infection and 1 h of gentamicin treatment, respectively. The results showed that cell association and internalization rate of the wild-type and its isogeneic strains were similar (Supplementary Figures S2A,B). In addition, to demonstrate whether NADase activity is required for GAS multiplication in HMEC-1 cells, the ribosomal *rpsL* promoter was utilized to drive an endogenous inhibitor of NADase, IFS, in the wild-type NZ131. The results demonstrated that overexpression of *ifs* in NZ131 (SW960) resulted in the similar phenotypes, including NADase and intracellular survival, compared to the *nga* mutant (**Figures 1E,F**).

**FIGURE 2:**
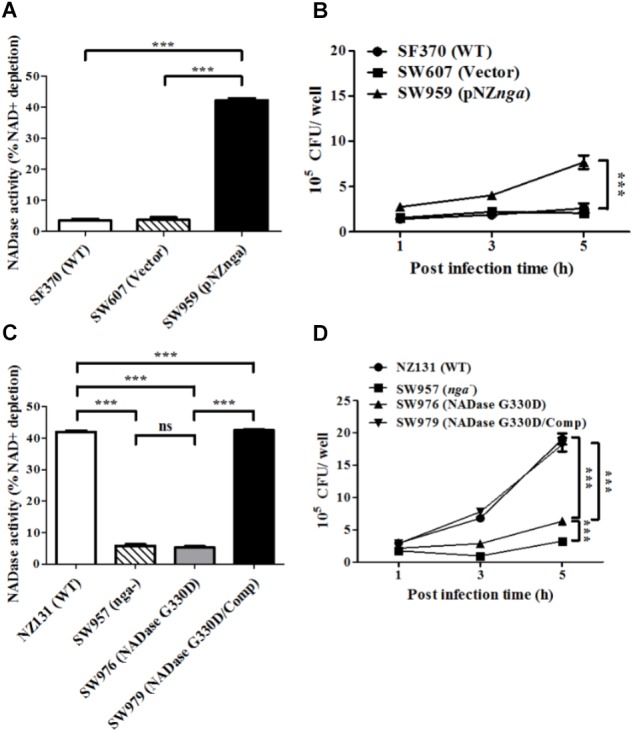
The enzymatic activity of NADase is required for the intracellular growth of GAS in endothelial cells. **(A,C)** NADase activities of different strains were determined by co-incubation with NAD^+^ and expressed as relative percentage compared to medium alone. **(B,D)** HMEC-1 cells were infected with strain SF370 with heterogeneous expression of *nga* at M.O.I. of 10 or strain SW976 (NADase G330D) at M.O.I. of 1. The intracellular viable bacteria were counted by CFU-based assay. The data represent the means ± SEM of at least three independent experiments. ns, not significant; ^∗∗∗^*p* < 0.001 (one- or two-way ANOVA).

Since the replacement of glycine by aspartic acid at 330 (G330D) in strain SF370 abrogates its NADase activity (Chandrasekaran et al., 2013; Zhu et al., 2015a), the *nga* of NZ131 (D330G) was heterogeneously expressed in SF370 (resulting in strain SW959) to restore its NADase activity (**Figure 2A**). The results showed that strain SW959 increased its intracellular growth by approximately 3-fold in HMEC-1 cells, compared to wild-type SF370 and vector control (SW607) strains (**Figure 2B**). The NADase G330D mutation in strain NZ131 (SW976) was also constructed to clarify the effect of NADase activity on intracellular multiplication of GAS in endothelial cells. Results showed that strain SW976 dramatically decreases the NADase activity and intracellular multiplication relative to the wild-type and NADase G330D complementary strains (SW979) in HMEC-1 cells (**Figures 2C,D**). Moreover, compared with the *nga* mutant, strain SW976 showed a significantly higher intracellular bacterial load (6.08 × 10^5^ cfu/ml versus 3.26 × 10^5^ cfu/ml) after 5 h of infection in HMEC-1 cells (**Figure 2D**). These results indicate that the enzymatic activity of NADase is required for GAS growth intracellularly.

### NADase Reduces Intracellular NAD^+^ Levels in Infected Endothelial Cells

**FIGURE 3:**
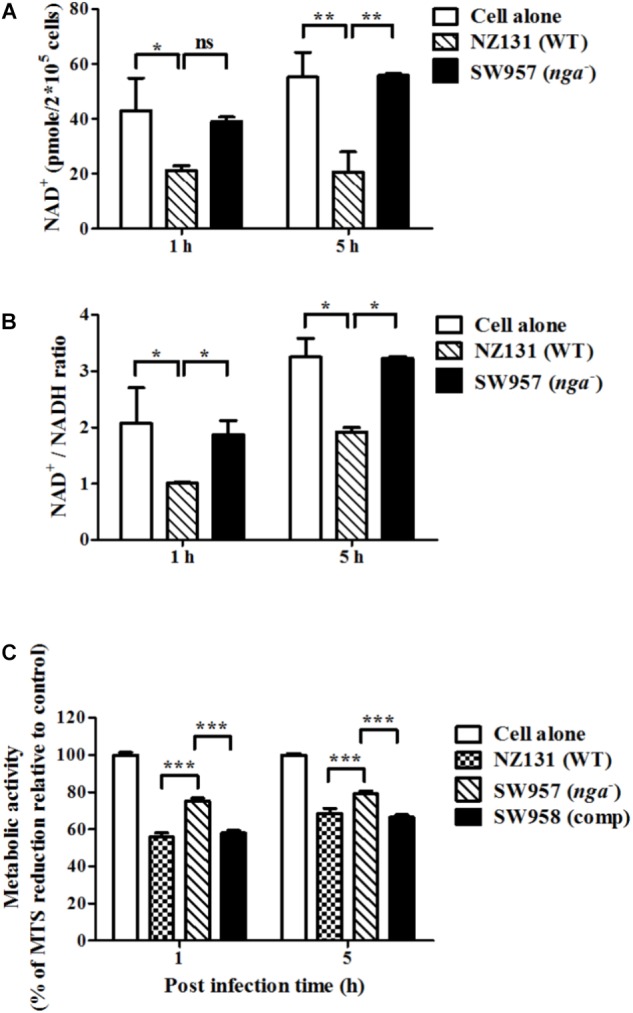
NADase depletes intracellular NAD^+^ content within infected endothelial cells. **(A)** The intracellular NAD^+^ content in endothelial cells after infection with NZ131 or the *nga* mutant. Intracellular NAD^+^ was extracted from GAS-infected HMEC-1 cells during infection and the content was analyzed by a NAD^+^/NADH quantification kit. **(B)** The NAD^+^/NADH ratio in endothelial cells after infection with NZ131 or the *nga* mutant. The NAD^+^/NADH ratio of cell lysates was calculated following a formula: [(NAD_total_ – NADH)/NADH]. **(C)** The cell viability of infected cells was analyzed by metabolic activity of mitochondria using the MTS assay. The data represent the means ± SEM of at least three independent experiments. ^∗^*p* < 0.05; ^∗∗^*p* < 0.01; ^∗∗∗^*p* < 0.001 (two-way ANOVA).

NADase is translocated into the cell cytosol to cleave intracellular NAD^+^ to cause energy depletion and promote GAS survival in infected cells (Michos et al., 2006; Ghosh et al., 2010; Chandrasekaran and Caparon, 2015). Accordingly, we determined whether NADase is responsible for the decrease of intracellular NAD^+^ content in GAS-infected endothelial cells. The results showed that a lower concentration of intracellular NAD^+^ was shown in NZ131-infected cells (21 and 20 pmole/2 × 10^5^ cells), when compared with non-infected (43 and 55 pmole/2 × 10^5^ cells) and *nga* mutant-infected cells (39 and 56 pmole/2 × 10^5^ cells) at 1 and 5 h of infection, respectively (**Figure 3A**). Moreover, the NAD^+^/NADH ratio was decreased in NZ131-infected HMEC-1 cells relative to non-infected and *nga* mutant-infected cells (**Figure 3B**). These results indicate that NADase can decrease the intracellular NAD^+^ and NAD^+^/NADH ratio of endothelial cells during infection.

NAD^+^ is a vital molecule involved in various metabolism in the cell (Canto et al., 2015). Previous studies have shown that NADase depletes cellular energy store to cause cell cytotoxicity and cell death (Bricker et al., 2002; Bastiat-Sempe et al., 2014; Chandrasekaran and Caparon, 2015). To clarify whether NAD^+^ decrease results in cell death of endothelial cells, the metabolic activity of mitochondria in GAS-infected cells was evaluate by the MTS assay. Results showed that *nga* mutant-infected cells have higher cell viability compared to NZ131- and complementary strain-infected cells (**Figure 3C**). These results indicate that NADase can decrease the intracellular NAD^+^ and NAD^+^/NADH ratio of endothelial cells, and lead to decrease cell viability during infection.

### NADase Prevents GAS-Containing Vacuoles Trafficking to Lysosomes in Endothelial Cells

**FIGURE 4:**
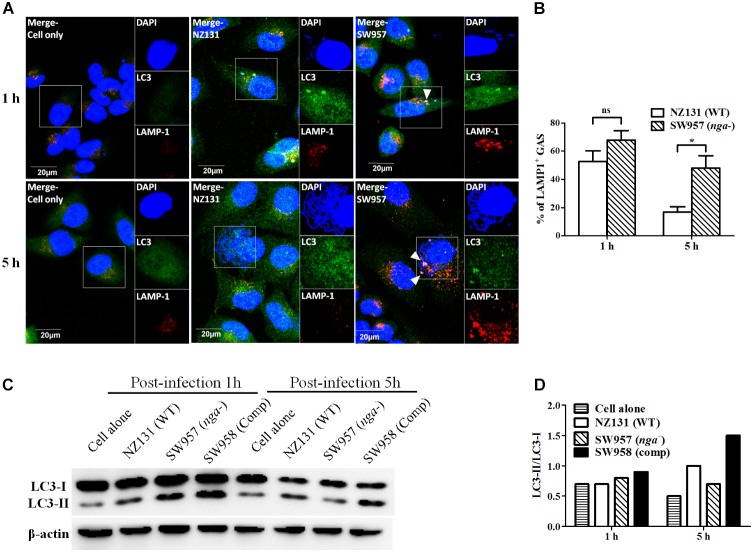
NADase prevents GAS-containing vacuole trafficking to lysosomes in endothelial cells. **(A)** The intracellular localization of GAS, LC3, and lysosome in endothelial cells after 1 and 5 h of infection. Confocal microscopy was used to observe the co-localization of GAS (DAPI, blue) with LC3 (Alexa 488, green) and LAMP-1 (Alexa 594, red) in HMEC-1 cells. Representative images are shown from three independent experiments. Scale bar = 20 μm. **(B)** The intracellular GAS associated with LAMP-1 at 1 and 5 h of infection were quantified from confocal microscopy images. At least 100 intracellular GAS were quantified for each time point in at least three independent experiments. The data represent the means ± SEM of at least three independent experiments. ns, not significant; ^∗^*p* < 0.05 (two-way ANOVA). **(C)** The LC3 conversion in endothelial cells after infection with NZ131 or the *nga* mutant. HMEC-1 cell lysates from uninfected (cell alone) or infected cells and the LC3 conversion were determined by the western blotting. **(D)** The expression of LC3-I and LC3-II were measured by densitometry analysis and was expressed as the ratio of LC3-II/LC3-I.

Several reports showed that NADase plays an important role in inhibiting lysosomal degradation in GAS-infected cells (O’Seaghdha and Wessels, 2013; Sharma et al., 2016). To better understand how NADase is utilized to enhance GAS survival in endothelial cells, confocal microscopy was used to observe the intracellular localization of bacteria, LC3-associated vacuoles, and lysosomes. The results showed that intracellular bacteria of both wild-type and *nga* mutant strains were co-localized with LC3-positive vacuoles (LC3 being a definitive marker of autophagosomes) after 1 h of infection in HMEC-1 cells (**Figure 4A**). However, the LC3 puncta was significantly decreased in NZ131-infected cells after 5 h of infection, compared to cells infected by the *nga* mutant (**Figure 4A**). In addition, the lysosomal-associated membrane protein 1 (LAMP-1), a membrane glycoprotein in lysosomes, was used to examine the co-localization of bacteria with lysosomes. The results showed that 53 and 68% of NZ131 and the *nga* mutant was associated with LAMP-1 after 1 h of infection, and 17 and 48% after 5 h of infection, respectively (**Figures 4A,B**). These observations indicate that NADase is able to inhibit trafficking of GAS to lysosomes.

The autophagosome-lysosome fusion is an effective process to eliminate invading GAS within infected cells (Sakurai et al., 2010; Nozawa et al., 2012). The LC3 conversion was examined by western blotting to confirm whether intracellular GAS can be trapped in autophagosome structures within endothelial cells. The results showed that the LC3-II rapidly accumulated in both the *nga* mutant and its complementary strains after 1 h of infection compared to the NZ131 strain (**Figure 4C**). However, the LC3-II signal and LC3-II/LC3-I ratio were lower in the *nga* mutant-infected cells than in cells infected by the NZ131 and complementary strains after 5 h of infection (**Figures 4C,D**). These results indicate that NADase could inhibit GAS-containing vacuoles fusing to lysosomes for degradation.

### The Acidification of GAS-Containing Vacuoles Is Inhibited by NADase in Endothelial Cells

**FIGURE 5:**
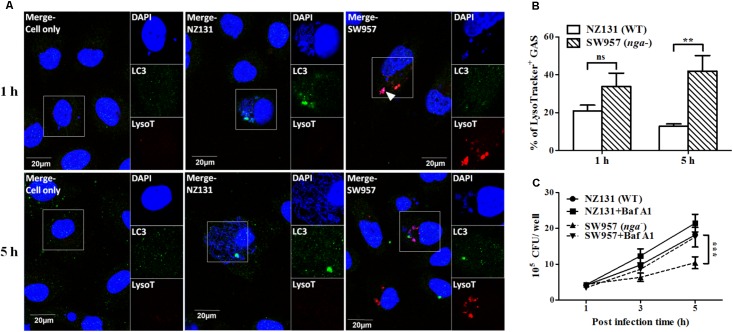
NADase prevents GAS-containing phagosome acidification in endothelial cells. **(A)** The intracellular localization of GAS (DAPI, blue), LC3 (Alexa 488, green), and acidification (acidotropic indicator LysoTracker Red DND-99, red) in endothelial cells were visualized by confocal microscopy after 1 and 5 h of infection. Representative images are shown from three independent experiments. Scale bar = 20 μm. **(B)** The percentage of intracellular GAS associated with LysoTracker was quantified at 1 and 5 h of infection. At least 100 intracellular GAS were quantified for each time point in at least three independent experiments. **(C)** The intracellular multiplication of NZ131 and *nga* mutant in endothelial cells after bafilomycin A1 treatment. HMEC-1 cells with or without pretreatment of bafilomycin A1 (100 nM) were infected with NZ131 or the *nga* mutant at M.O.I. of 1 and intracellular viable bacteria were counted by CFU-based assays. The data represent the means ± SEM of at least three independent experiments. ns, not significant; ^∗∗^*p* < 0.01; ^∗∗∗^*p* < 0.001 (two-way ANOVA).

An acidified environment in the mature autophagosome is required for the lysosome-mediated killing of intracellular bacteria (Mehrpour et al., 2010; Huang and Brumell, 2014). NADase has been shown to prevent acidification of GAS containing vacuoles and promote GAS survival in infected cells (Bastiat-Sempe et al., 2014). Therefore, we further used the lysotracker, a fluorescent dye for staining acidic organelles in live cells, to investigate if NADase inhibits autophagosomal acidification in endothelial cells. Confocal images and quantitative results showed that more *nga* mutant cells were co-localized with lysotracker (34 and 42% after 1 and 5 h of infection, respectively) than those of NZ131 (21 and 13% after 1 and 5 h of infection, respectively) (**Figures 5A,B**). Next, vacuolar H^+^-ATPase was inhibited by bafilomycin A1 to prevent lysosomal acidification. The results showed that the number of intracellular bacteria of the *nga* mutant was significantly increased after 5 h of infection under bafilomycin A1 treatment, compared to untreated cells (**Figure 5C**). These results clearly point out that NADase plays a crucial role in the inhibition of acidification of GAS-containing vacuoles in endothelial cells.

## Discussion

**FIGURE 6:**
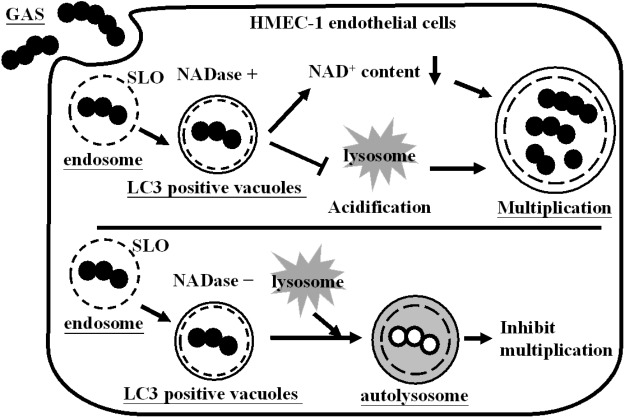
Influence of NADase on GAS survival in endothelial cells. GAS is internalized into cells through endocytosis to form the endosomes. However, endosomal membrane can be damaged by SLO action and further encapsulated in LC3-positive vacuoles. NADase is translocated into cell cytosol, where it depletes intracellular NAD^+^ and inhibits lysosome-mediated acidification to enhance GAS multiplication inside endothelial cells.

The endothelial cells line the interior surface of blood vessels to mediate vascular permeability and leukocyte trafficking. Moreover, endothelial cells play an important role in GAS infections (Amelung et al., 2011; Lee and Liles, 2011; Ochel et al., 2014). We previously showed that GAS resides in autophagosome-like vacuoles, but insufficient acidification of vacuoles allows efficient multiplication of bacteria within endothelial cells (Lu et al., 2015). In this study, we not only found that NADase depletes intracellular NAD^+^, but also inhibits acidification of GAS-containing vacuoles, enhancing intracellular multiplication in endothelial cells (**Figure 6**).

The co-localization of LC3 puncta with lysosomes was observed in GAS-infected endothelial cells at 1 h of infection, but *nga* mutant-infected cells showed much greater than wild-type NZ131-infected cells at 5 h of infection (**Figures 4A,B**). In contrast, the level of LC3-II was higher in wild-type NZ131-infected cells at 5 h of infection than *nga* mutant-infected cells (**Figures 4C,D**). Autophagy is a vital defense mechanism that requires lysosome fusion to facilitate clearance of intracellular pathogens in infected cells (Nakagawa et al., 2004; Barnett et al., 2013; O’Seaghdha and Wessels, 2013; Huang and Brumell, 2014). However, if autophagic flux is blocked at the step of autophagosome-lysosome fusion, the level of LC3-II is accumulated in the cell (Klionsky et al., 2016), indicating that although autophagy can be induced by GAS, the step of autophagosome fusion with the lysosome is blocked by NADase in endothelial cells.

A previous study showed that sufficient acidification not only represses the transcription level of *nga* and *slo*, but also arrests the GAS growth *in vitro* (Lu et al., 2015). The mature acidified vacuole (pH < 5.0) is required to activate lysosomal hydrolase that contributes to resisting bacterial growth in infected cells (Yates and Russell, 2005; Mehrpour et al., 2010). In this study, we showed that NADase inhibits lysosome-mediated acidification to allow bacterial multiplication (**Figures 5A,B**). Similar results were also reported in macrophages and keratinocytes, where NADase was shown to enhance SLO-mediated membrane damage, which contributes to inhibiting acidification of GAS-containing vacuoles and promoting GAS survival intracellularly (Logsdon et al., 2011; Bastiat-Sempe et al., 2014). These evidences clearly indicate that a battle between the bacterial factors and autophagic defense mechanisms to determine GAS survival within infected cells.

Lower intracellular NAD^+^ level, NAD^+^/NADH ratio, and cell viability were observed in NZ131-infected cells compared to non-infected and *nga* mutant-infected cells (**Figure 3**). In epithelial cells and macrophages, NADase has been shown to cleave NAD^+^ to induce intracellular energy depletion, which results in cytotoxicity and programmed necrosis of infected cells (Michos et al., 2006; Ghosh et al., 2010; Chandrasekaran and Caparon, 2015; Sun et al., 2015; Chandrasekaran and Caparon, 2016). NAD^+^ is a vital molecule involved in various physiological functions and plays an important role in pathogen infection (Mesquita et al., 2016). Increasing evidences indicated that NAD^+^ homeostasis is tightly regulated in all cells and modulates autophagy responses via many NAD^+^-consuming enzymes (Ng and Tang, 2013; Zhang et al., 2016). The poly-ADP-ribose polymerase-1 (PARP-1) is an important NAD^+^-consuming enzyme involved in multiple physiological processes, including autophagic activation (Munoz-Gamez et al., 2009). Chandrasekaran and Caparon (2015) recently reported that NADase activity can modulate PARP-1 activation to trigger programmed cell death. These results suggest that energy depletion may contribute to impair cell defense mechanisms against GAS infection in infected cells. However, how NADase modulates host factors to regulate autophagic maturation for intracellular multiplication of GAS in endothelial cells requires further study.

Several epidemiological studies reported that epidemic M1 and M89 GAS strains harbor an amino acid substitutions at residue 330 of NADase, which increases enzymatic activity, inducing severe tissue injury and enhancing global dissemination (Turner et al., 2015; Zhu et al., 2015a,b, 2016; Chochua et al., 2017). In this study, we demonstrated that NADase activity is required for intracellular GAS multiplication in endothelial cells (**Figure 1**). Furthermore, cells infected with the NADase G330D strain have higher bacterial loads than *nga* mutant-infected cells (**Figure 2D**), suggesting that inactive NADase still has a minor role in the intracellular multiplication of GAS in endothelial cells. Recent studies showed that NADase activity depletes intracellular energy store to trigger programmed necrosis in epithelial cells known as metabolic catastrophe (Chandrasekaran and Caparon, 2015). On the other hand, NADase without enzymatic activity has shown to induce cell programmed necrosis by JNK1 and PARP-1 activation, and enhance inflammation in epithelial cells and macrophages (Chandrasekaran and Caparon, 2016; Hancz et al., 2017). These studies not only indicated that the enzymatic activity of NADase provides a strategy for GAS survival, but also that NADase protein without NADase activity can still modulate cellular responses of infected cells to enhance GAS pathogenesis.

In summary, we conclude that NADase and its enzymatic activity are required for promoting the intracellular multiplication of GAS through intracellular NAD^+^ depletion and inhibiting lysosome-mediated acidification in endothelial cells. The ability of NADase to mediate efficiently multiplication of GAS in endothelial cells may facilitate GAS invasion of bloodstream and systemic infection.

## Author Contributions

Y-SL, CC-N, P-JT, S-YW, C-CL, and J-JW conceived and designed the study. C-LH, H-MH, S-YH, P-XZ, Y-SL, and J-JW acquisition and analysis of the experiments. C-LH and J-JW wrote the manuscript.

## Conflict of Interest Statement

The authors declare that the research was conducted in the absence of any commercial or financial relationships that could be construed as a potential conflict of interest.
