# Supplementary material for: NAD-Glycohydrolase Depletes Intracellular NAD+ and Inhibits Acidification of Autophagosomes to Enhance Multiplication of Group A Streptococcus in Endothelial Cells
Source: Front Microbiol. 2018 Aug 3;9:1733. doi: 10.3389/fmicb.2018.01733 (PMC6085451; doi:10.3389/fmicb.2018.01733)
Supplement: Supplementary file 1 [file Table_1.docx]

**SUPPLEMENTARY MATERIALS**

**FIGURE S1. The transcription of *nga*, *slo*, and *ifs* in the *nga* mutant were quantified by qRT-PCR.** RNAs were extracted from different strains at the exponential phase of growth in TSBY. Real-time RT-PCR (qRT-PCR) was performed with specific primers for (A) *nga* (B) *ifs* (C) *slo*. The relative expression of each gene was compared to NZ131. Data represent the means ± SEM of at least three independent experiments. ***, *p* < 0.001 (one-way ANOVA)

**FIGURE S2. NADase does not affect the association and internalization of GAS to endothelial cells.** HMEC-1 cells were infected with wild type NZ131 or the *nga* mutant at M.O.I of 1 for 30 min and the unbound bacteria were removed by PBS. The cell-associated bacteria were counted by CFU-based assays (A). Gentamicin was used to kill extracellular bacteria after 30 min of infection. The internalized bacteria were counted by CFU-based assays (B). The data represent the means ± SEM of at least three independent experiments. ns, not significant.

**SUPPLEMENTARY TABLE 1. Primers used in this study**

| Name | Sequence (5’ to 3’)^#^ | Comment |
| --- | --- | --- |
| *nga*+*ifs*-F | CGGAATTCAACGGATGACTATTGT | for *nga* knockout and NADase G330D mutant construction |
| *nga*+*ifs*-R | CGGGATCCCTTCATACCTTTTTAT | for *nga* knockout and NADase G330D mutant construction |
| NADase G330D-F | GACCAAATTGAAAATATAAAAGATGTCGATAGCGGAAAATATAG | for NADase G330D mutant construction |
| NADase G330D-R | CTATATTTTCCGCTATCGACATCTTTTATATTTTCAATTTGGTC | for NADase G330D mutant construction |
| *nga*-qF | TGTTGCTATTGCTTTGGCTG | for qRT-PCR of *nga* |
| *nga*-qR | TTGAGCCGTCTAATGTGTGC | for qRT-PCR of *nga* |
| *ifs*-qF | AGAAATGTCAAATAGCGGTCAAG | for qRT-PCR of *ifs* |
| *ifs*-qR | CCATAGCCTCTCTAATATGCGC | for qRT-PCR of *ifs* |
| *slo*-qF | AACAAACCAGACGCGGTAGT | for qRT-PCR of *slo* |
| *slo*-qR | GCAGGAAGCGTATTACCACCA | for qRT-PCR of *slo* |

^#^ Engineered restriction endonuclease sites are underlined.
