# Supplementary figures and images for: NAD-Glycohydrolase Depletes Intracellular NAD+ and Inhibits Acidification of Autophagosomes to Enhance Multiplication of Group A Streptococcus in Endothelial Cells
Source: Front Microbiol. 2018 Aug 3;9:1733. doi: 10.3389/fmicb.2018.01733 (PMC6085451; doi:10.3389/fmicb.2018.01733)

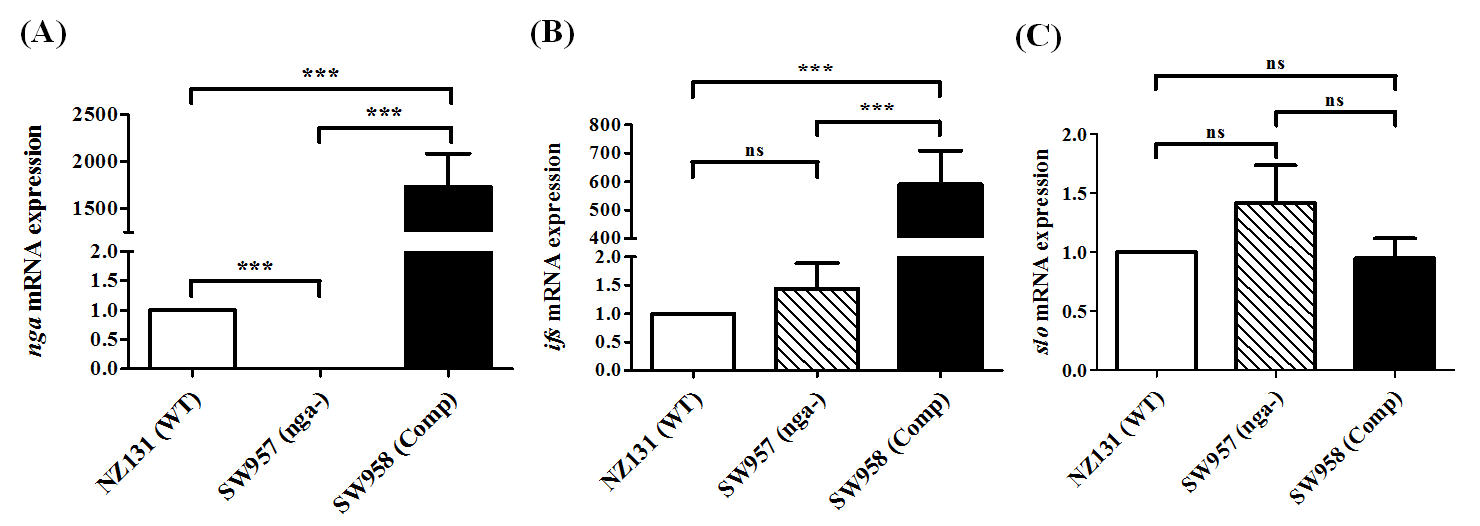

Supplement: Supplementary file 2 [file Image_1.tif]

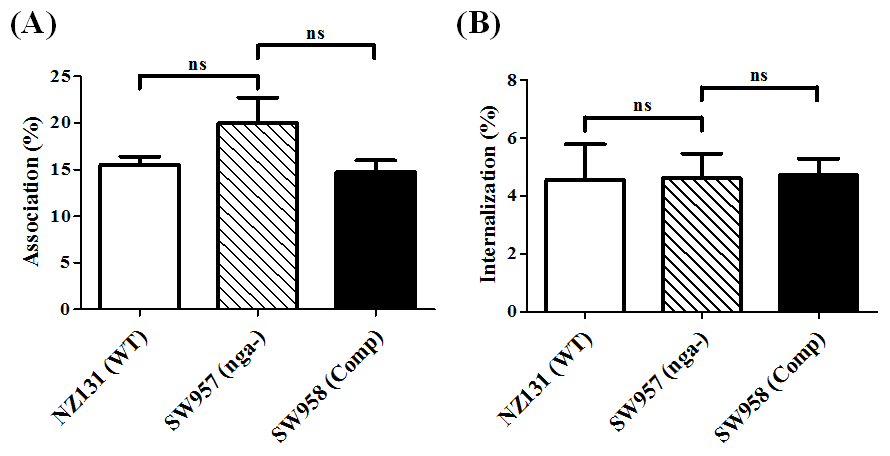

Supplement: Supplementary file 3 [file Image_2.tif]
